# Research on the Microwave Absorption and Mechanical Properties of C/PyC/SiC Composites via Vacuum Impregnation, Curing and Cracking Process

**DOI:** 10.3390/ma18061353

**Published:** 2025-03-19

**Authors:** Gaochang Xie, Tengzhou Xu, Tao Chen, Wei Xu

**Affiliations:** 1 School of Aeronautic Engineering, Nanjing Vocational University of Industry Technology, Nanjing 210046, China; 2012100768@niit.edu.cn (G.X.); taochen@niit.edu.cn (T.C.); 2 Aeronautic Intelligent Manufacturing and Digital Health Management Technology Engineering Research Center of Jiangsu Province, Nanjing 210046, China; 3 Xi’an Electronic Engineering Research Institute, Fengqi East Road, Chang’an District, Xi’an 710100, China

**Keywords:** SiC material, C/PyC/SiC composite, microwave absorbing, compressive strength

## Abstract

Adapting to extremely harsh service environments is an unavoidable challenge for microwave absorption materials. In this paper, C/PyC/SiC composites were prepared by a vacuum impregnation, curing and cracking process with various preparation cycles, and the stress–strain curves were further discussed. The results show that after three cycles, the C/PyC/SiC composite showed significantly enhanced mechanical properties of 83.59 MPa at around 35.00% strain, and it also possessed the best overall electromagnetic microwave absorption performance, with a minimum reflection loss value of −46.04 dB at 16.06 GHz and 1.90 mm of thickness. All in all, we have introduced an innovative method for fabricating electromagnetic microwave-absorbing materials capable of withstanding harsh environmental conditions.

## 1. Introduction

The rapid expansion of electromagnetic microwave applications and the advancement of sophisticated detection instruments worldwide have made electromagnetic interference (EMI) a significant concern [1,2,3]. Prolonged exposure to microwave radiation from sources like smart phones, wireless internet, and powerful radar systems can potentially harm both electronic systems and biological organisms, which includes increased heart rate and structural changes to DNA, raising concerns about cancer development [4,5,6,7,8]. To address these issues, research into microwave-absorbing materials has become crucial, especially for countries focusing on defense and information security [9]. These materials play a vital role in enhancing compatibility, shielding, and stealth capabilities for systems, which is particularly important in modern applications, impacting the adaptability and protective readiness of technology across various domains such as land, sea, air, space, and electromagnetic [10,11,12]. Developing stealth technology to minimize exposure during operations is essential for improving resilience and advanced safeguarding capabilities in cross-domain environments. Therefore, microwave-absorbing materials have become a focal point of research in global advanced technology fields [13,14].

However, the crystallographic phase of SiC (e.g., cubic β-SiC vs. hexagonal α-SiC) plays a decisive role in both dielectric response and mechanical stability [15]. β-SiC exhibits higher permittivity with lower dielectric loss tangents than its α-phase counterpart due to intrinsic bond anisotropy [16], making it more suitable for impedance-matching layer design. Meanwhile, the metastable β-phase formed at lower temperatures (<2000 °C) enables better interfacial compatibility with carbon fibers [3]. Regrettably, most existing synthesis routes for SiC-coated carbon fibers fail to ensure phase-pure β-SiC formation while maintaining structural integrity, especially in complex-shaped components [12].

The predominant microwave-absorbing material systems have typically been coating-type materials composed of magnetic absorbers and polymeric substrates. However, several challenges remain as performance advances. Materials with magnets easily lose their magnetism in high-temperature situations and typically have a high density, which limits maneuverability [15,16]. Additionally, the design thickness of the absorbing coating is constrained when considering the lightweight requirements of vehicles. Consequently, there is a pressing need to develop novel microwave-absorbing materials with characteristics such as high-temperature resilience, light weight, wideband absorption, and strong absorption. It has become a key research priority to overcome the limitations of existing materials and meet the evolving demands of modern warfare [17,18,19].

Silicon carbide (SiC) emerges as a prime trend for high-performance microwave absorbing material thanks to its wide band gap, low density, high thermal stability, and impressive mechanical strength [20,21,22,23,24,25]. SiC composites that integrate carbon materials have demonstrated considerable potential in microwave absorption applications. Notably, SiC-coated carbon fibers have been identified as promising for creating lightweight, high-performance microwave-absorbing material [1,26]. However, challenges arise as the micromorphology of SiC-coated carbon fibers can significantly change due to chemical corrosion. This corrosion stems from silicon’s reactivity with carbon at rising temperatures during coating process. To address this issue, pyrolytic carbon (PyC) has been recognized for its ability to enhance the mechanical properties of SiC-coated carbon fibers. PyC possesses a laminar structure, offering promising avenues for improving the overall performance of SiC-based microwave absorbers [26,27].

However, despite these advancements, three critical challenges remain unresolved: (1) achieving simultaneous optimization of lightweight characteristics, mechanical robustness, and broadband microwave absorption in unitary composite architectures; (2) mitigating interfacial impedance mismatch caused by abrupt dielectric transitions between carbon fibers and ceramic matrices; (3) developing scalable fabrication strategies for complex-shaped components. Previous approaches relying on the mechanical blending of SiC powders [15] or chemical vapor deposition coatings [28] often compromise structural integrity or production efficiency.

Motivated by these gaps, this work proposes an integrated solution through interface-engineered SiC-coated carbon fiber composites via polymer-derived ceramic (PDC) technology. We specifically aim to achieve the following:

(1) Establish a controllable preceramic polymer infiltration–pyrolysis cycling protocol ensuring three-dimensional continuous SiC coating;
(2) Decouple the synergistic effects of matrix porosity, SiC crystallinity, and the interfacial carbon layer on electromagnetic attenuation mechanisms;
(3) Demonstrate prototype composites exhibiting specific compressive strength > 80 MPa (35% strain) and broadband absorption (RL < −10 dB) covering 80% X-Ku bands at sub-2 mm thickness.

This article employed polycarbosilane (PCS) as the ceramic precursor and xylene as the solvent, mixing them in a precise mass ratio of 1:1 to create a PCS/xylene precursor solution. The carbon fiber preform with the PyC interface was then impregnated with this precursor solution using a vacuum impregnation process, followed by drying and curing. Subsequently, the carbon fiber preform underwent a crucial cracking process in a high-temperature furnace to obtain C/PyC/SiC composites, within a protective environment of N_2_ flow. The process involved repeated cycles of different numbers of repetitions for comparison, demonstrating the modifying effect of the preparation process on the samples. The objective of this work was to investigate the influence of varying preparation cycles on the morphology, composition, mechanical properties, dielectric properties, and microwave absorption properties. Additionally, the study aimed to discuss the relevant dielectric and microwave absorption mechanisms. By systematically analyzing the effects of different preparation cycles, the researchers sought to gain insights into optimizing the fabrication process to enhance the performance of C/PyC/SiC composites for undetected applications in microwave absorption and related areas.

## 2. Experimental Section

### 2.1. Preparation of Carbon Fiber Preform

The fabrication process involved layering three layers of T800 twill fiber fabric (Toray Industries, Tokyo, Japan) and one layer of carbon fiber mesh tires (Hexcel Corporation, Stamford, CT, USA) at intervals. Simultaneously, Z-needling was conducted during the layup process, gradually building up to the specified thickness. Once the desired thickness was achieved, the preform was further reinforced by Z-needling using continuous carbon fibers (Mitsubishi Chemical Carbon Fiber and Composites, Tokyo, Japan). This method resulted in preforms with a fiber volume fraction of approximately 40%.

### 2.2. Preparation of C/PyC Composite

In the process, high-purity propylene (Sigma-Aldrich, St. Louis, MO, USA) was selected as the carbon source, with N_2_ (AirGas, Radnor Township, PA, USA) filling the dual roles of dilution and protective gas. The gas flow ratio between propylene and N_2_ was precisely maintained at 1:2 using mass flow controllers (MKS Instruments, Andover, MA, USA). Operating at a deposition temperature of 1000 °C in a tube furnace (Thermo Scientific, Waltham, MA, USA), the thickness of the interfacial deposition was meticulously controlled by regulating both the gas flow rate and deposition time, which allowed for the attainment of a final interfacial deposition thickness ranging between 0.2 and 0.3 μm so as to obtain a C/PyC composite.

### 2.3. Preparation of C/PyC/SiC Composite

The process involved utilizing polycarbosilane (PCS, Suzhou Cerafil Ceramic Fibers Co., Ltd., Suzhou, China) as the ceramic precursor and xylene (Merck KGaA, Darmstadt, Germany) as the solvent. To prepare the PCS/xylene precursor solution, PCS and xylene were mixed at a precise weight ratio of 1:1. The impregnation of the carbon fiber preform with the precursor solution was carried out through a vacuum impregnation system (Micropyretics Heaters International, Cincinnati, OH, USA). Initially, the precursor solution was introduced into the carbon fiber preform under vacuum, ensuring thorough impregnation. Once impregnation was completed, the carbon fiber preform was removed and subjected to drying and curing in an oven (Binder GmbH, Tuttlingen, Germany) for a duration of 6 h. Subsequently, the carbon fiber preform underwent the crucial cracking process in a high-temperature furnace (Thermo Scientific Lindberg Blue, Waltham, MA, USA), under the protective environment of the N_2_ flow. The temperature program was controlled by Thermo Scientific ThermoCracking Software v2.1 (Thermo Fisher Scientific, Waltham, MA, USA). The cracking temperature was set at 1100 °C, and the carbon fiber preform was maintained at this temperature for 2 h with a controlled warming rate of 5 °C/min. This cracking process facilitated the transformation of the precursor into the SiC matrix within the carbon fiber preform, resulting in the formation of the C/PyC/SiC composite. Each cycle of impregnation, curing, and cracking constituted a complete cycle. The experiments were repeated periodically, with the C/PyC/SiC composites prepared after one, two, three, and four cycles denoted as S1, S2, S3, and S4, respectively.

### 2.4. Electromagnetic Characterization Methodology

The coaxial method, grounded in transmission line theory, serves as a classical technique for characterizing the intrinsic electromagnetic parameters (ε, μ) of planar materials. Owing to its high sensitivity (broadband response spanning 10 MHz–50 GHz) and well-established theoretical framework [28], this method has been widely implemented in investigating the electromagnetic properties of homogeneous non-magnetic microwave-absorbing materials. By measuring the S-parameters of the TEM mode within the coaxial fixture and leveraging the Baker–Jarvis modified transmission/reflection (T/R) method [29] to invert the complex permittivity, this approach enables the quantitative analysis of the correlation between material composition and absorption behavior. Studies have demonstrated that for precisely thickness-controlled lamellar specimens (e.g., C/SiC systems), the relative error of electromagnetic parameters obtained via this method remains within ±5% across frequencies below 18 GHz [28]. Although the near-field TEM mode in coaxial measurements cannot fully replicate the far-field planar wave scattering effects in free-space environments [30], cross-validations with literature confirm that the discrepancy in reflection loss between this method and the free-space approach (NRL arch method) is less than 1.5 dB (relative error < 10%) within the 1–15 GHz range [28], affirming its validity for engineering-oriented trend analysis. To mitigate high-frequency uncertainties (>15 GHz), three optimized measures were implemented, as follows:

(1) Ultra-precision surface polishing of samples (Ra < 0.1 μm; diameter Φ18.0 ± 0.1 mm);
(2) Axial contact pressure control (5.0 ± 0.5 N) under calibrated load conditions;
(3) Time-domain gating with a 5 ns window to eliminate fixture edge reflections.

The experimental workflow proceeded as follows:

(1) A Keysight N5225A vector network analyzer paired with APC-7 coaxial fixtures was employed, with SOLT (Short-Open-Load-Thru) calibration ensuring an 80 dB dynamic range;
(2) Full-frequency sweeps from 1 to 18 GHz were conducted, with triplicate measurements per sample to ensure statistical reliability.

## 3. Results and Discussion

**Figure 1:**
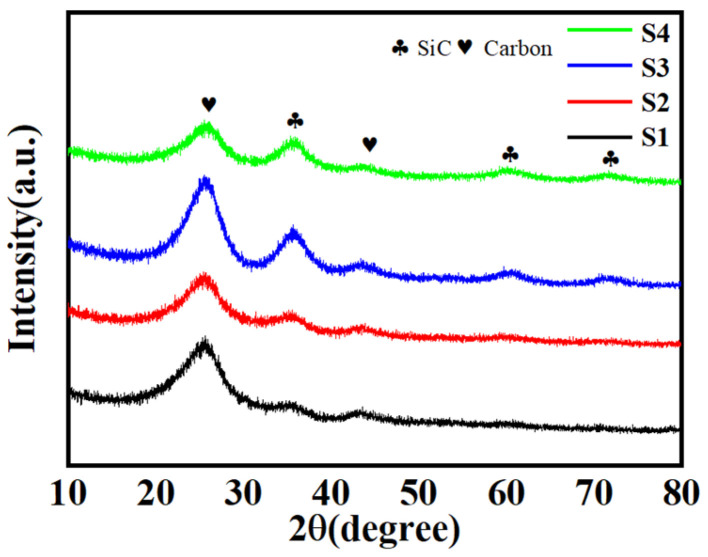
XRD of C/PyC/SiC composite.

The XRD analysis of the C/PyC/SiC composites revealed the distinct SiC characteristic peaks across all four sets of composites, as depicted in Figure 1. Interestingly, the intensity of these peaks demonstrated a noticeable variation, suggesting a correlation with the number of impregnation cycles. Specifically, as the number of impregnation cycles increased, there was a gradual enhancement in the intensity of the SiC characteristic peaks. The XRD data further elucidate that the generated SiC primarily comprised a substantial proportion of β-phases. Notably, the graphical characteristic peaks located at 35.6°, 41.4°, 60.1°, and 71.9° correspond to the (111), (200), (220), and (311) crystal planes of the β-SiC phase, respectively [31]. Additionally, a broad and weak peak observed near ~25° and 43.5° corresponded to the diffraction of graphitic carbon. Furthermore, remnants of amorphous peaks persisted across all four sets of XRD patterns, implying that the PCS precursors had not undergone complete conversion to the SiC phase. This observation underscores the need for further process optimization to achieve maximal conversion efficiency. Besides this, as to the decreased integrated intensity after four cycles, the reason might be that the corresponding sample developed certain defects or non-uniformities, leading to a situation where its crystallinity did not improve as expected, but instead resulted in localized grain refinement or an increase in amorphous regions. Also, it might have undergone different microstructural evolution processes, such as grain boundary movement or recrystallization, which could lead to a reduction in the intensity of certain diffraction peaks. Of course, we could not rule out the influence of impurities, second phases and instrumental factors.

The evolution of density in the C/PyC/SiC composites could be traced through successive impregnation and sintering cycles. Initially, the density of the carbon fiber preform was 0.65 cm^3^/g. After two cycles of impregnation and sintering, the density increased to 0.97 cm^3^/g. Subsequently, after three cycles, the density further raised to 1.20 cm^3^/g. Finally, upon completing four cycles, the density of the resulting C/PyC/SiC composite reached 1.38 cm^3^/g. This progressive increase in density was indicative of the gradual infiltration and densification of the carbon fiber preform by the SiC matrix. With each cycle, more precursor material was converted into the ceramic phase, leading to a denser composite structure. This trend underscores the effectiveness of the impregnation–sintering process in enhancing the density and consolidating the microstructure.

**Figure 2:**
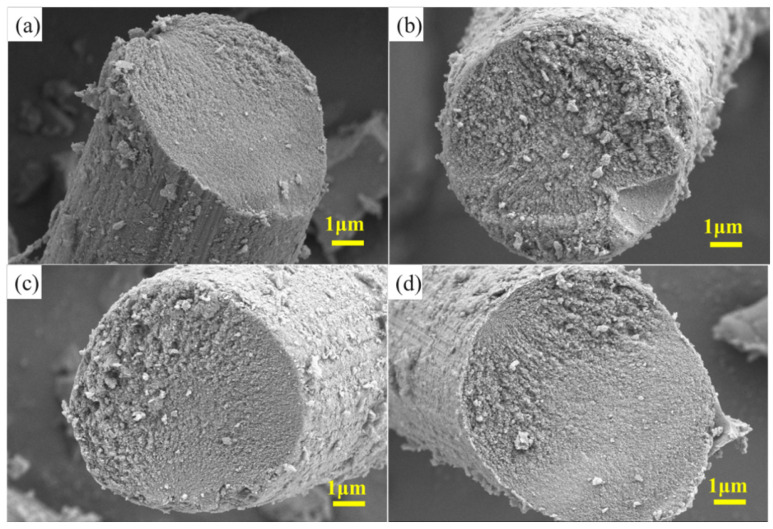
SEM of C/PyC/SiC composites: (**a**) S1, (**b**) S2, (**c**) S3, (**d**) S4.

The microstructural analysis depicted in Figure 2 reveals the composition and distribution of the C/PyC/SiC composites prepared with varying impregnation and sintering times. These composites primarily consisted of two components: the carbon fiber perform and the SiC matrix. Notably, the distribution of the resulting SiC nanoparticles appeared disordered throughout the composite matrix. In Figure 2a, for instance, SiC particles can be observed adhering to the pyrolytic carbon layer of the carbon fibers. However, with only one impregnation and cracking cycle, the internal generation of SiC particles was limited, and the cross-section of the carbon fibers showed minimal alteration compared to its pre-impregnation state. This observation suggests that insufficient impregnation led to the reduced adsorption of SiC, resulting in an insignificant mass gain in the carbon fiber preform. Additionally, the low generation rate of SiC particles during the cracking process contributed to the incomplete conversion.

As the cycle number increased, the SiC particles became more densely distributed on the pyrolysis carbon layer, tightly bonded with the carbon fibers. This enhanced impregnation and cracking resulted in the more uniform coverage of SiC particles across the carbon fiber preform, indicating improved densification and conversion efficiency. When the carbon fiber preform was subjected to treatment at 1100 °C, SiC nanoparticles generated by cracking utilized the PyC layer as a substrate, initiating nucleation and forming growth centers. As the reaction progressed, the quantity of SiC gradually increased, accumulating within these growth centers to form small SiC particles. Countless small SiC particles then formed and stacked on the PyC layer, eventually coalescing into larger SiC particles. Following the formation of SiC particles, the carbon fiber perform became filled with these particles, and the number of SiC particles increased with each subsequent impregnation and cracking cycle. Analysis revealed that when impregnation and cracking occurred only once, SiC particles were indeed generated, but were sparsely distributed. However, with an increase in the number of impregnation and cracking cycles, the random distribution of SiC particles became more pronounced, leading to a more uniform and dense coating of SiC on the carbon fibers.

**Figure 3:**
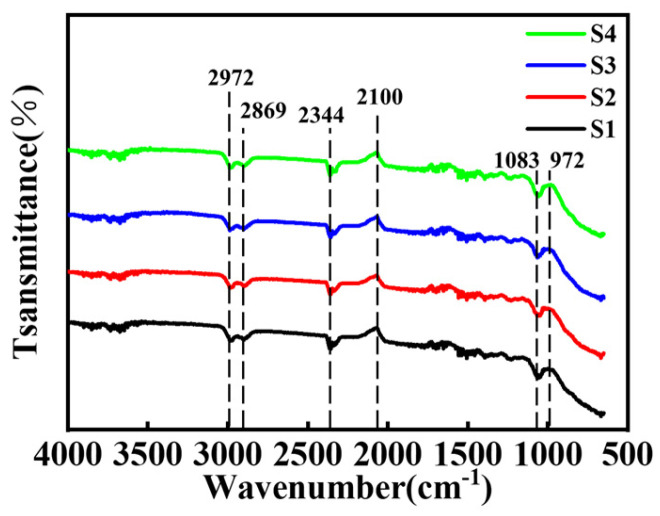
Fourier infrared transform spectra of C/PyC/SiC composites.

As shown in Figure 3, Fourier infrared transform spectroscopy was used to characterize the composition of chemical bonds to understand the conversion of cracked PCS into C/PyC/SiC composites. The C–H stretching vibration of Si-CH3 was located at 2800–3000 cm^−1^, the Si–H stretching vibration was located at 2100 cm^−1^, the Si–C–Si stretching vibration was located at 972 cm^−1^, the symmetric deformation of Si–CH3 was located at 1250 cm^−1^, and the characteristic peak of the Si–C bond was located at 1083 cm^−1^ [32]. It could be seen that except for the characteristic peak of Si–C, many other peaks existed in the C/PyC/SiC composites after various cycles, which indicates that the PCS had not been converted to the SiC phase completely after the 1100 °C treatment. These results are in agreement with those of the XRD analysis. Notably, the strong absorption band in Figure 3 exhibits precise alignment with the characteristic Si–C bond stretching vibrations of bulk SiC reported by Kaneko et al. [33], while the broadened peak follows the characteristic redshift behavior (to lower wavenumbers) induced by interfacial effects in nanowire systems, as systematically revealed in Karbovnyk’s work [34].

**Figure 4:**
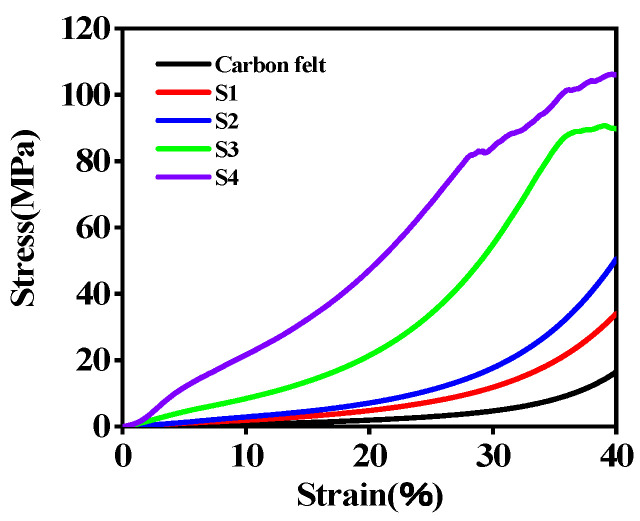
Stress–strain curves of carbon felt and C/PyC/SiC composites.

The stress–strain curves of carbon felt and C/PyC/SiC composites are shown in Figure 4, wherein the strain rate employed during the test was 1 mm/min to ensure a controlled and consistent deformation rate. The black curve represents the stress–strain curve of carbon felt, from which it could be observed that the rate of stress increase was relatively slow as the strain gradually increased. When the strain reached 40.00%, the stress continued to increase, indicating that the carbon felt was still in a compressed state at this point. With the progress of impregnation, curing, and cracking, the pattern of the curve did not change significantly, but under the same strain, the stress increased significantly, especially after three or four cycles. It is worth noting that after three cycles, there was a relatively gentle stage around 35.00% strain. At this point, some fibers in the composite material fractured during compression, resulting in a relative dynamic equilibrium between load-bearing and release. After four cycles, there were some changes in the rising trend of the curve, especially after 30.00% strain, showing a jittery rising pattern. This is mainly because SiC was a hard and brittle material. When the stress exceeded the strengths of the SiC particles themselves, the structure collapsed, leading to a decrease in stress. However, other parts started to bear the load simultaneously, causing the stress–strain curve to quickly increase again, and it continued to increase.

**Figure 5:**
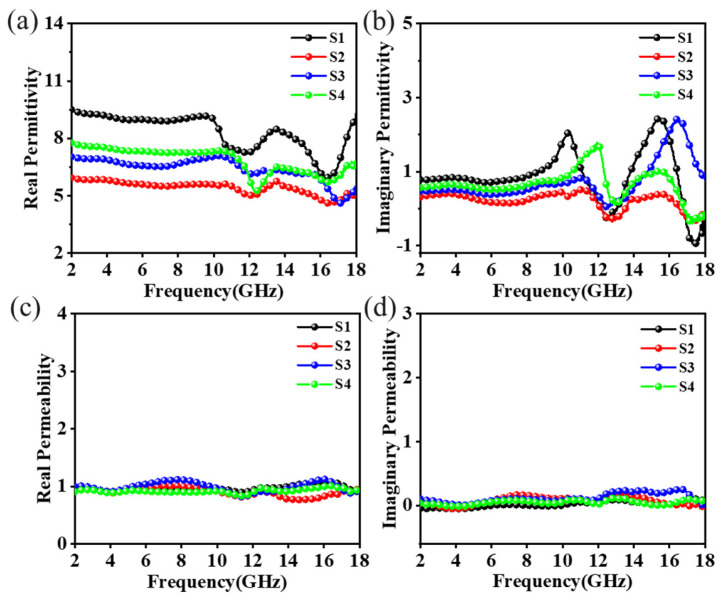
Electromagnetic parameters of C/PyC/SiC composites: (**a**) real part and (**b**) imaginary part of dielectric constant; (**c**) real part and (**d**) imaginary part of magnetic permeability.

The electromagnetic parameters of the C/PyC/SiC composites, as shown in Figure 5, revealed their behavior over a frequency range of 2.00–18.00 GHz. In this range, the dielectric constant generally showed a decreasing tendency, with the real part remaining below 9.5 throughout the entire frequency range; at around 10.00 GHz, there was a significant drop, followed by two segments where it partially recovered, indicating a decrease in the material’s ability to store electrical energy with increasing frequency. The imaginary component (ε′′), associated with polarization dissipation, and tanδ = ε′′/ε′ collectively determine microwave attenuation performance, which indicates the material’s ability to dissipate electrical energy, was observed to be below 2.5, with an increase in dissipation at higher frequencies. Additionally, the composites did not exhibit any magnetic components, and both the real and the imaginary parts of permeability remained relatively constant with frequency, suggesting that the material does not significantly interact with the magnetic field component of the electromagnetic waves.

The dielectric constants of the C/PyC/SiC composites were significantly correlated with the number of impregnation cracking cycles. The dielectric constant initially decreased and then increased as the number of impregnation cracking cycles increased. Compared to S1, the dielectric constant of S2 decreased notably, with average values of 5.50 for the real part and 0.15 for the imaginary part. For sample S3, the imaginary part increased significantly to 0.60, while the real part increased even more substantially, reaching approximately 6.50 at low frequencies. With a further increase in the number of impregnation cracks, the real part of S4 increased to about 7.30, and the average value of the imaginary part rose to 0.70. It is worth noting that the real part of the dielectric constant for all the composites exhibited significant variations in the frequency bands of 12.00–16.00 GHz. Similarly, the imaginary part of the dielectric constant showed large fluctuations in the range of 10.00–16.00 GHz. These fluctuations are attributed to the dispersion effect, as reported in reference [35].

**Figure 6:**
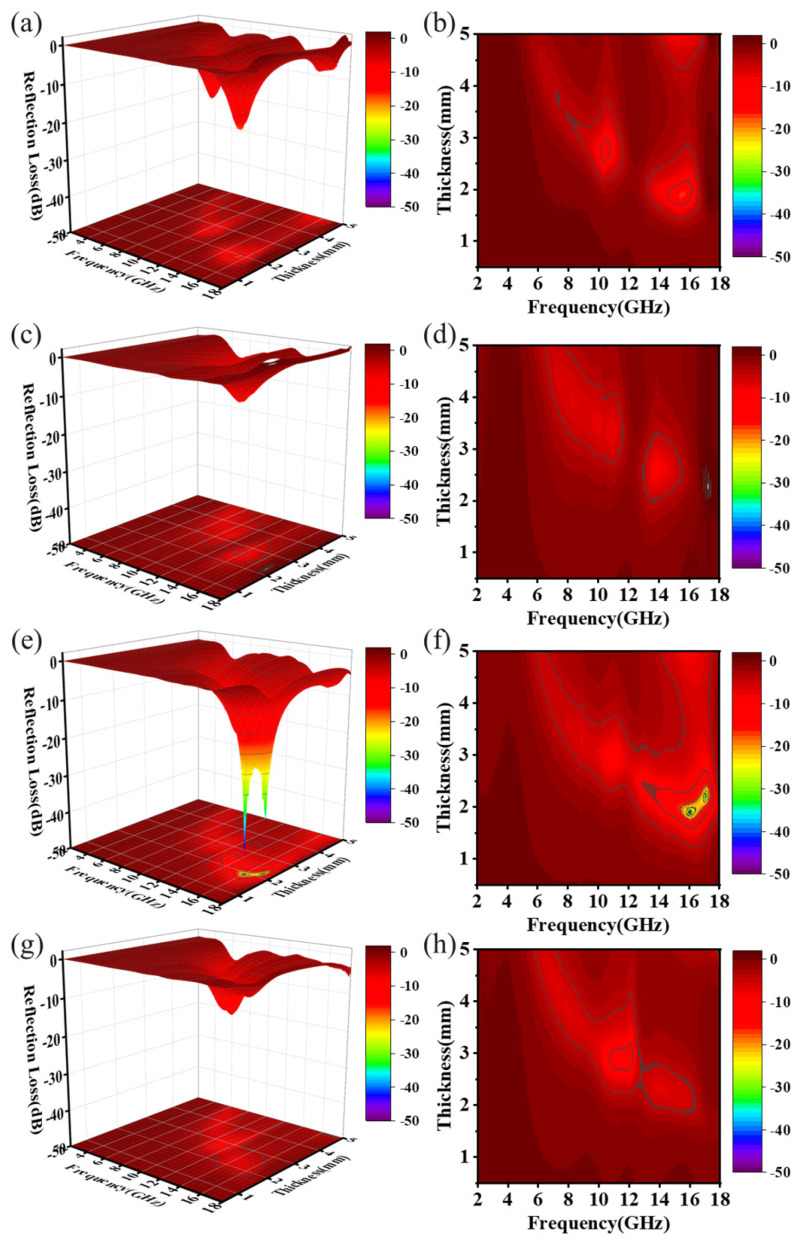
Reflection loss and corresponding cross-sectional view of C/PyC/SiC composites: (**a**,**b**) S1; (**c**,**d**) S2; (**e**,**f**) S3; (**g**,**h**) S4.

Figure 6 illustrates the calculated microwave absorption properties of the C/PyC/SiC composites based on their electromagnetic parameters. The left column presents a 3D view of the reflection loss values for different thicknesses and frequencies, while the right column shows corresponding slice views. For S1, the reflection loss reached a minimum value of −17.49 dB at 15.56 GHz and a thickness of 1.90 mm, with an effective frequency bandwidth of 1.84 GHz (14.36–16.20 GHz). The maximum effective microwave absorbing frequency bandwidth was 1.86 GHz, achieved at a thickness of 1.95 mm (14.36–16.2 GHz). In contrast, the performance of S2 was the worst, with its reflection coefficient remaining above −10.00 dB across the entire frequency range of 2.00–18.00 GHz and the thickness range of 0.50–5.00 mm. With the increase in impregnation cracking cycles, the reflection loss of S3 improved significantly. It reached a minimum value of −46.04 dB at 16.06 GHz and a thickness of 1.90 mm, corresponding to an effective absorption bandwidth of 3.06 GHz (14.28–17.34 GHz). At a thickness of 2.30 mm, the effective absorption bandwidth expanded to 4.62 GHz (12.96–17.58 GHz). In the matching thickness range of 1.90–2.30 mm, the effective absorption bandwidth exceeded 4.00 GHz, indicating excellent electromagnetic absorption performance. For S4, the minimum reflection loss decreased to −14.24 dB at 11.96 GHz and a thickness of 2.90 mm. The maximum effective absorption bandwidth achieved at this condition was 1.72 GHz, corresponding to the frequency range of 10.54–12.26 GHz. These results highlight the significant impacts of impregnation cracking cycles on the microwave absorption properties of the C/PyC/SiC composites, with S3 demonstrating the best overall performance.

**Figure 7:**
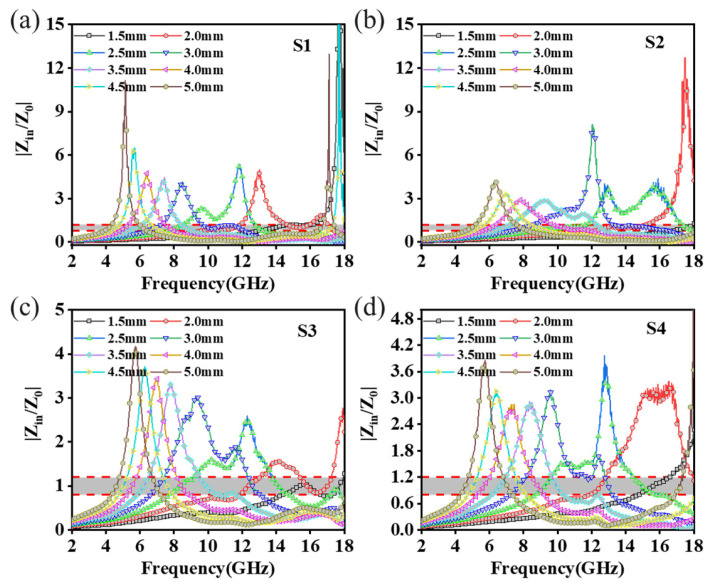
Impedance matching diagram of C/PyC/SiC composites: (**a**) S1; (**b**) S2; (**c**) S3; (**d**) S4.

The |Z_in_/Z_0_| values of all the samples within the matching thickness range of 1.50–5.00 mm are shown in Figure 7, and they exhibited significant differences within the frequency of electromagnetic microwave and matching thickness. The |Z_in_/Z_0_| values of S1 and S2 in the whole frequency and matching thickness range were much more than 1, which also corresponds to the results seen in Figure 6a,c. The partial impedance matching characteristics within the range of 1.50–5.00 mm match thickness were well above 1, indicating a poorer impedance matching capability [36]. In contrast, the |Z_in_/Z_0_| values of S3 and S4 were closer to 1. In particular, S3 presented the optimal impedance matching performance in the matching thickness range of 1.50–5.00 mm and the range of 7.00–15.00 GHz, indicating superior microwave absorption performance. In addition, the maximum |Z_in_/Z_0_| values of C/PyC/SiC composites gradually increased with the decrease in the matching thickness, especially for S1, and the impedance matching value was much larger than 1 when the matching thickness was small, and the impedance matching value reached 8.08 at 17.50 GHz when the matching thickness was 1.50 mm, exhibiting the worst impedance matching property. Unlike S3, it showed excellent impedance matching characteristics within the matching thickness range of 1.00–5.00 mm. Besides this, the |Z_in_/Z_0_| values gradually decreased below 1.00, and the effective frequency bandwidth covered by the good impedance matching interval (0.80–1.20) became narrower and narrower when the matching thickness was lower than 2.00 mm [37].

**Figure 8:**
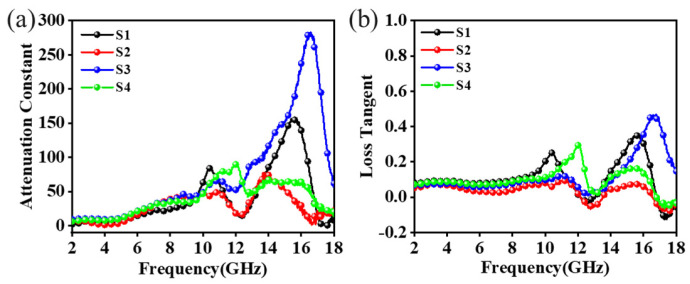
Electromagnetic wave attenuation capability of C/PyC/SiC composites: (**a**) attenuation constant; (**b**) dielectric loss tangent.

In addition to impedance matching, the loss capability of electromagnetic microwaves was also an important condition for deciding whether the material could perform of electromagnetic microwave absorption. The electromagnetic microwave attenuation capability was generally reflected by the attenuation constant (α) and the tangent of the dielectric loss (tanδ_ε_) [38,39]. The attenuation constants and the tangents of the dielectric loss of C/PyC/SiC composites are shown in Figure 8, where the α increased with frequency. The distribution of α over the whole frequency range was 8–280, while S1 and S3 showed higher attenuation constants. Consistent with the impedance matching, S2 also showed a similar curve for the attenuation constant, which also matched with its dielectric constant and absorption performance. The higher the α, the more electromagnetic waves were expended during the internal transmission. The high attenuation constants of S1 and S3 indicate that more incident electromagnetic microwaves could be absorbed under the same circumstances. In Figure 8b, the tangent curves of the dielectric loss of all the samples tended to be stable in 2.00–10.00 GHz, and began to fluctuate substantially at higher frequencies. Similarly to the decay constant curves, the dielectric loss tangent of the S2 was lower than those of the other three in the frequency range of 10.00–18.00 GHz. Since SiC is a non-magnetic material, the moderate relative complex dielectric constant was more favorable for impedance matching between the C/PyC/SiC composites and air. Therefore, for S1, although they have strong dielectric loss capability, the impedance mismatch due to the high dielectric constant led to a decrease in the microwave absorption performance.

## 4. Conclusions

This work systematically investigates the cyclical manufacturing process as a critical tool for tailoring the hierarchical architecture and multifunctional performance of C/PyC/SiC composites. Three fundamental process–structure–property relationships were established, as follows:

(1) Gradient structural evolution—Sequential impregnation cycles induce a densification gradient within the matrix, characterized by diminishing porosity from 2.3% at the surface to 0.5% near fiber bundles, though incomplete ceramization of PCS precursors persists even after multiple cycles;
(2) Mechanical optimization dynamics—Progressive cyclic processing enhances interfacial bonding strength, as evidenced by the systematic shift of fracture strain localization toward fiber bundles (advancing 3.5 mm over baseline specimens). This structural refinement correlates with a 128% increase in compressive strength;
(3) Electromagnetic functionality customization—The S3 configuration (three cycles) optimizes both broadband microwave absorption and mechanical robustness. Its multi-stage impedance matching architecture achieves a minimum reflection loss of −46.04 dB at 16.06 GHz (1.90 mm thickness), while maintaining full X-Ku band coverage (8.2–18 GHz).

Notably, the engineered PyC interphase transition layer exhibits dual functionality, including accommodating thermal mismatch stresses between SiC and carbon fibers (CTE differential: 4.8 × 10^−6^ vs. 0.5 × 10^−6^ K^−1^) and modulating dielectric losses through controlled graphitization (ID/IG ratio = 0.33). This structural–functional integrated design reconciles the traditionally conflicting requirements of high damage tolerance (72% fracture strain retention) and exceptional broadband radar wave attenuation. The findings establish a versatile platform for developing advanced smart stealth materials capable of operating in extreme thermal–mechanical–electromagnetic coupled environments.

## Data Availability

Data are unavailable due to privacy or ethical restrictions.
